# Pericardial Synovial Sarcoma Masquerading as Hemangioma: A Diagnostic Challenge

**DOI:** 10.7759/cureus.81076

**Published:** 2025-03-24

**Authors:** Kesar Prajapati, Fnu Samaksh, Poornima Jaiswal Charpuria, Nisarg Desai, Sibasis Sahoo

**Affiliations:** 1 Internal Medicine, New York Medical College, Metropolitan Hospital Center, New York, USA; 2 Cardiology, U. N. Mehta Institute of Cardiology and Research Centre (UNMICRC), Ahmedabad, IND

**Keywords:** echocardiography, hemangioma, pericardial sarcoma, pericardial synovial sarcoma, tumor

## Abstract

Pericardial synovial sarcoma (PSS) is a rare primary malignant tumor of the heart with an unclear prognosis. We present the case of a 26-year-old male patient with no significant medical history who presented with New York Heart Association (NYHA) Class II dyspnea and chest pain. Echocardiography and cardiac MRI revealed a large pericardial mass (93 × 70 × 45 mm) with hemorrhagic effusion and imaging features suggestive of hemangioma, including well-defined vascular channels and contrast enhancement. Histopathological analysis following thoracotomy showed spindle cell proliferation without classic features of malignancy (e.g., nuclear atypia and high mitotic activity), supporting the initial diagnosis of spindle cell hemangioma. However, six months later, a recurrent mass excision and immunohistochemistry (IHC) confirmed SS (transducin-like enhancer of split 1/FMS-like tyrosine kinase 1 (TLE1/FLT1) positive). Surgical resection was attempted but was not feasible due to the extensive involvement of critical cardiac structures. The patient was started on chemotherapy with ifosfamide and doxorubicin but succumbed to systemic complications within a year. This case underscores the diagnostic challenge of PSS and highlights the critical role of IHC and molecular diagnostics in distinguishing it from benign mimics, even when initial histopathology is inconclusive.

## Introduction

Pericardial masses are diagnostically challenging, encompassing both benign and malignant lesions. Primary pericardial tumors are exceedingly rare, accounting for less than 1% of all cardiac neoplasms, with metastatic involvement being far more common [1]. Among primary malignancies, synovial sarcoma (SS) is a particularly rare and aggressive subtype, typically arising in periarticular soft tissues but rarely reported in the pericardium [2]. SS’s predilection for younger adults and its insidious presentation, often mimicking benign conditions like pericardial effusion or hemangioma, further complicate timely diagnosis [3]. 

Primary pericardial synovial sarcoma (PSS) is extremely rare, with fewer than 20 reported cases [4]. Differential diagnoses include spindle cell hemangioma, mesothelioma, lymphoma, and metastatic tumors, each with distinct features like diffuse thickening, systemic symptoms, or known primaries, necessitating careful evaluation to avoid misdiagnosis [5].

Contrast-enhanced MRI and PET-CT are valuable for differentiating SS from benign lesions. MRI can reveal heterogeneous signal intensity, necrosis, and invasive features, while PET-CT demonstrates hypermetabolic activity suggestive of malignancy, potentially prompting earlier molecular and histopathological investigations [6]. The diagnostic challenge of PSS arises from its histopathological overlap with benign vascular tumors, such as spindle cell hemangioma, and nonspecific imaging findings. Initial evaluations, including pericardial fluid cytology, often lack specificity, necessitating advanced immunohistochemical and molecular analyses [7]. Immunohistochemical markers, such as transducin-like enhancer of split 1 (TLE1) and FMS-like tyrosine kinase 1 (FLT1), are reliable for diagnosing SS, though their underutilization in initial workups may delay definitive diagnosis [8]. Molecular testing for the SYT-SSX fusion transcript, using reverse transcription polymerase chain reaction (RT-PCR) or fluorescence in situ hybridization (FISH), remains the gold standard, with nearly 100% specificity [9].

This case report describes a 26-year-old male patient diagnosed with a benign spindle cell hemangioma following pericardiocentesis and excision. Six months later, he experienced a recurrence with rapid progression to lethal SS. This report aims to highlight the challenges of diagnosing and treating PSS, emphasizing the importance of considering malignancy in the differential diagnosis of pericardial masses. Early molecular diagnostics are crucial for accurate diagnosis, guiding treatment, and preventing delays, as demonstrated in this case.

## Case presentation

**Figure 1:**
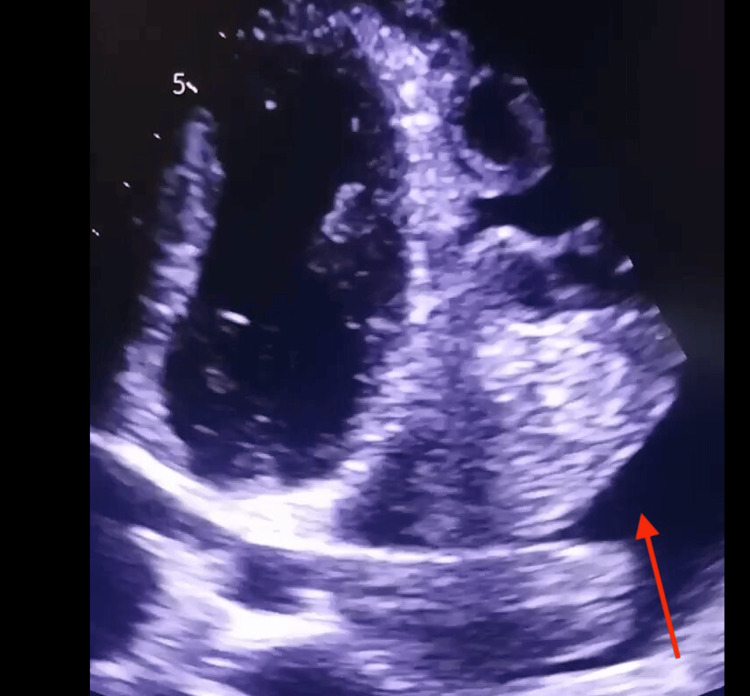
Apical four-chamber echocardiographic view showing a heterogeneous, echogenic mass in the pericardium (red arrow), with an adjacent anechoic space suggestive of pericardial effusion.

A 26-year-old male patient of Indian origin with no significant medical history presented with symptoms of shortness of breath, chest tightness, fatigue, and atypical chest pain for one month. Symptoms progressively worsened, with no history of trauma, infections, or occupational exposures. He experienced mild limitations in performing daily activities, consistent with New York Heart Association (NYHA) Class II dyspnea. On examination, the patient was tachycardic with a heart rate of 110 beats per minute (bpm), but his blood pressure was normal at 130/76 mmHg, and his respiratory rate was 20 breaths per minute. Oxygen saturation was 98% on room air, indicating adequate oxygenation. Systemic examination revealed muffled heart sounds, clear bilateral airways, no jugular venous distention, and no pedal edema. The echocardiogram showed a large pericardial effusion with an echogenic mass in the pericardial cavity near the left ventricle and atrium (Figure 1).

**Figure 2:**
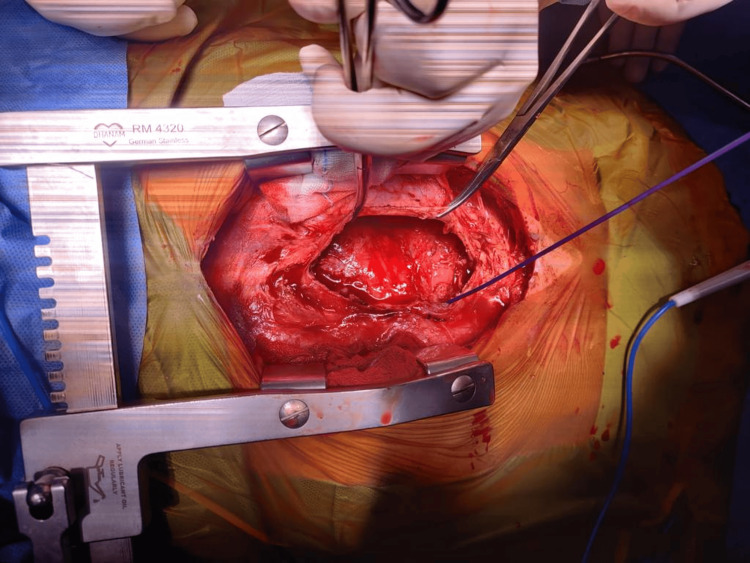
Intraoperative view of an open thoracotomy with a surgical retractor in place, exposing the pericardial cavity.

**Figure 3:**
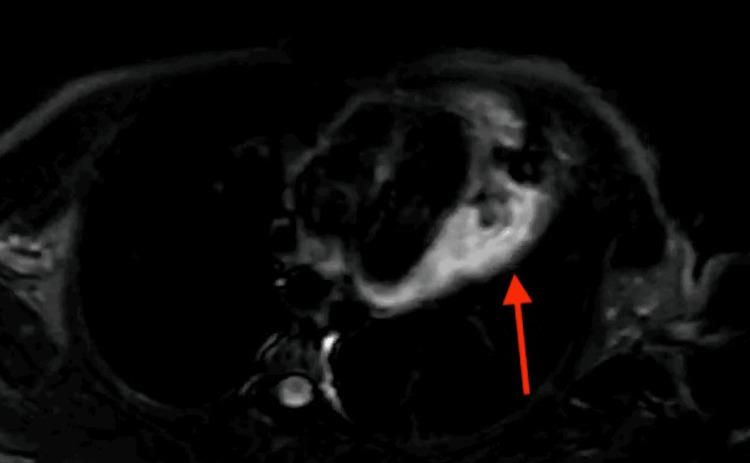
Cardiac MRI demonstrating a pericardial mass (red arrow) with hyperintense signal on late gadolinium enhancement.

**Figure 4:**
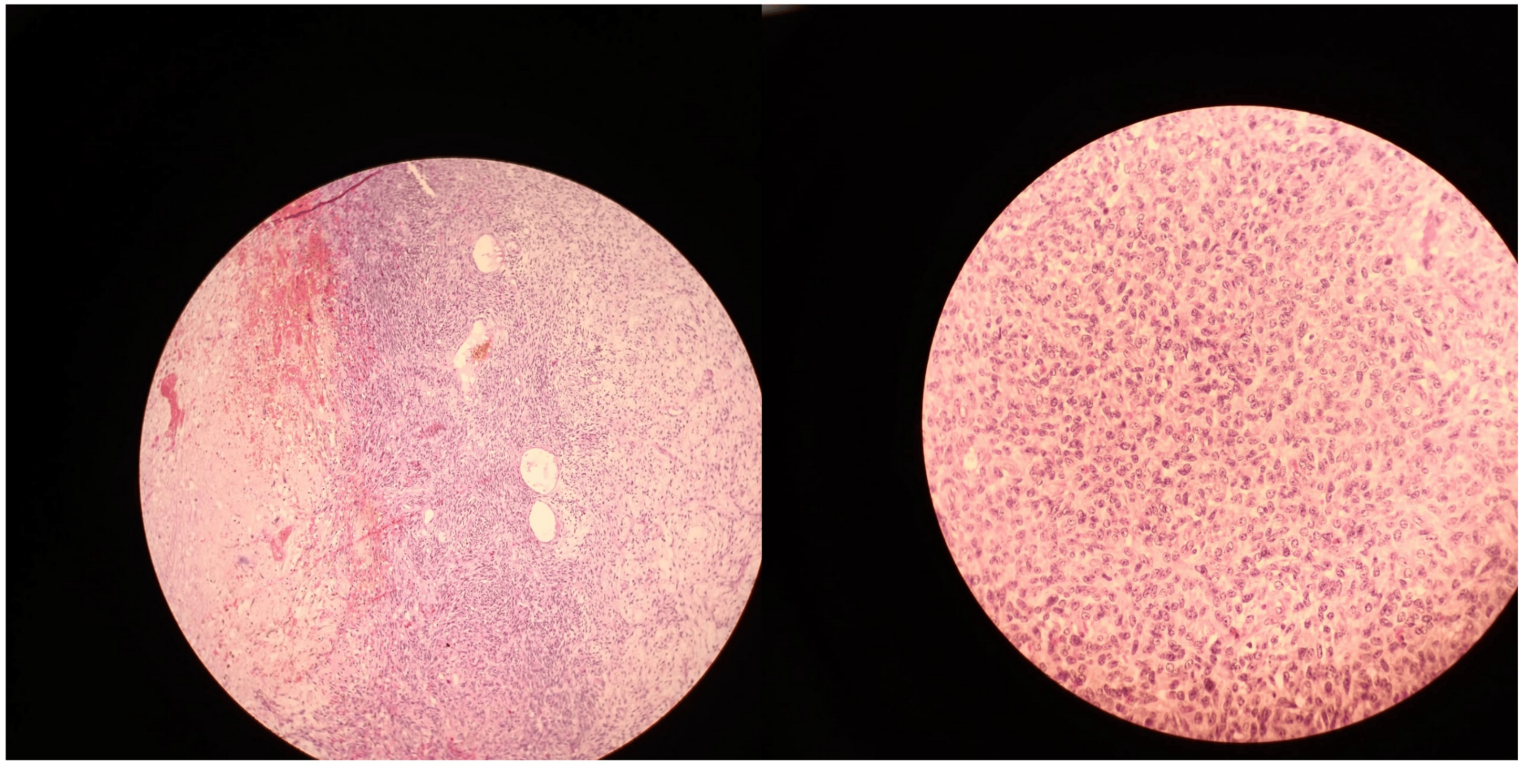
Histopathological image of pericardial sarcoma stained with hematoxylin and eosin. The low-magnification view shows congested vascular channels lined by flattened endothelial cells with foci of spindle cell proliferation.

The patient underwent pericardiocentesis, and the drained fluid was sent for histopathological examination. The results revealed hemorrhagic fluid with an RBC count of 8-10 per high-power field, a total count of 180/mm^3^, protein 4.5 gm/dL, and glucose 108 mg/dL. Cytological and microbiological analyses were unremarkable. The differential count showed 70% lymphocytes and 30% neutrophils, consistent with a nonspecific inflammatory response. Cardiac MRI post-drainage revealed a well-defined, irregular mass measuring 93 x 70 x 45 mm in the pericardial cavity overlying the left lateral margin of the left atrium and mid-basal ventricle adherent to the visceral pericardium. The mass appeared inhomogeneously iso- to hyperintense on T1-weighted imaging (T1WI) and hyperintense on T2-weighted imaging (T2WI). Short tau inversion recovery (STIR) sequences showed patchy areas of diffusion restriction, raising concern for a more aggressive lesion, though these findings were not definitive for malignancy (Figure 2). The patient underwent open thoracotomy for surgical excision of the mass (Figure 3). Histopathological examination revealed congested vascular channels lined by flattened endothelial cells with foci of spindle cell proliferation and minimal mitotic activity (Figure 4), suggestive of a spindle cell variant of pericardial hemangioma without signs of malignancy. No atypical features (e.g., nuclear pleomorphism or high mitotic activity) were noted initially to suggest malignancy. Following the excision of the benign mass and symptomatic support, the patient’s symptoms improved, and he was discharged after a few days.

However, six months later, the patient presented to the emergency department with a recurrence of similar symptoms. Echocardiography and MRI revealed a recurrent mass, now located in the right anterior mediastinum (pericardial mass or mediastinal mass?), compressing the right heart. The mass was found to completely cover the right atrium, superior and inferior vena cavas, ascending aorta, and part of the pulmonary artery. It also partly covered the anterior wall of the right ventricle. Immunohistochemistry (IHC) of the recurrent mass confirmed primary synovial cell sarcoma, positive for the TLE1 and FLT1 markers. Biopsy confirmed the diagnosis of SS, although molecular testing (e.g., SS18-SSX fusion) was not detailed. The patient was started on chemotherapy with ifosfamide and doxorubicin. The hematology-oncology and cardiothoracic surgery teams were consulted, and surgical excision was attempted. However, due to the extensive involvement of critical cardiac structures, complete resection was not feasible, and chemotherapy was initiated. The patient succumbed to systemic complications, likely due to metastatic disease or treatment toxicity.

## Discussion

Heart tumors are rare, often discovered incidentally as cardiac masses, typically indicating thrombi or neoplasms in specific clinical settings. The incidence of cardiac tumors ranges from 0.001% to 0.03%, with the majority being benign. Malignant tumors make up about one-fourth of these cases [10]. PSS is an extremely rare cardiac tumor, with only 36 reported cases as of 2018 [4]. This rarity limits our understanding of the condition, although it is known to have a poor prognosis. SS is an uncommon malignant tumor most often found in children and adolescents, with a 10-year survival rate of 0%-20% [4]. The tumor is believed to originate in synovial tissue and is classified into three histological subtypes: biphasic (epithelial and spindle cells), monophasic (spindle cells only), and poorly differentiated (small round cells) [11]. Primary pericardial tumors are rare, with pericardial cysts and lipomas being the most common benign tumors and mesothelioma being the most common primary malignant tumor. Other malignant neoplasms include various sarcomas and lymphomas, among which SS is extremely rare. PSS typically presents with dyspnea and signs of cardiac tamponade due to large pericardial effusions, typically affecting men around 35 years old [2].

The morbidity rate in young individuals with PSS is notably high, with the pericardium being the most commonly affected site. In the case presented, the tumor was also located in the pericardium. The clinical signs of PSS are often vague and nonspecific, with chest pain and dyspnea being the most common symptoms. Our patient experienced chest tightness, shortness of breath, and fatigue before hospitalization. Early diagnostic imaging revealed significant pericardial effusion. A study conducted in China identified malignant tumors and tuberculosis as the leading causes of pericardial effusion [12]. However, in this case, pericardiocentesis and analysis of the pleural effusion did not confirm the presence of malignant cells. It is important to note that in about 25% of malignant pericardial effusion cases, tumor cells may be absent from the fluid sample. Thus, despite the absence of malignant cells in the pericardial effusion, the possibility of a malignant pleural effusion should not be ruled out entirely [13].

The initially missed diagnosis in the case, where the absence of tumor cells and related markers in the pleural fluid was noted, can be attributed to several factors. First, the initial symptomatic treatment led to significant improvement in the patient's symptoms, which may have contributed to a false sense of resolution and delayed further investigation. Additionally, the absence of tumor cells and related markers in the pleural fluid likely resulted in the misinterpretation of the condition as benign or nonmalignant [14]. No follow-up imaging was initially planned due to symptom resolution and benign histopathology. However, routine imaging every 3-6 months would have been beneficial to monitor for recurrence, given the tumor’s size and aggressive features [15]. While initial pericardial fluid analysis did not reveal malignant cells, additional cytological techniques, such as cell block preparation or FISH, could have increased the diagnostic yield [15]. However, these were not performed initially due to the low suspicion of malignancy at the time. Over the next six months, the tumor progressed, leading to the recurrence of symptoms. Echocardiography revealed a mass compressing the right heart and the root of the great arteries, prompting further investigation. The eventual diagnosis of SS was confirmed through immunohistochemical analysis, which identified specific markers such as TLE1 and FLT1. Histopathological examination of the recurrent mass confirmed the monophasic subtype of SS, characterized by spindle cells. This case underscores the necessity of considering malignancy in the differential diagnosis of pericardial masses and highlights the value of molecular and cytogenetic studies, such as SYT-SSX fusion analysis, for accurate diagnosis [14]. The rapid recurrence of PSS, even after complete excision, illustrates its aggressive nature and tendency for local invasion and metastasis. This is consistent with prior reports suggesting a poor prognosis for this type of tumor. The aggressive behavior and poor prognosis of SS are well-documented, with frequent local recurrence and metastasis despite aggressive treatment modalities [16].

The cornerstone of treatment for SS is surgical excision, followed by systemic chemotherapy and, in selected cases, radiation therapy. In this case, the initial surgery failed to prevent recurrence due to the malignant nature of the tumor. Additionally, the tumor in our case (93 x 70 x 45 mm) was significantly larger than those reported in the literature, making complete resection more challenging [17]. Anthracycline-based chemotherapy (e.g., doxorubicin) combined with ifosfamide remains the standard first-line treatment for advanced disease. However, these treatments often face limitations in preventing recurrence and managing systemic complications, especially in larger tumors [18]. Emerging therapies provide hope for better outcomes. Targeted molecular inhibitors such as pazopanib, which targets vascular endothelial growth factor (VEGF) receptors, have shown some efficacy in SS. Immunotherapies, including adoptive cell transfer using engineered T-cell receptors targeting antigens like NY-ESO-1, PRAME, and MAGE-A4, are also being explored. Additionally, immune checkpoint inhibitors (e.g., pembrolizumab) are under investigation, although their efficacy in SS remains limited [17].

The rapid recurrence of PSS, even after complete excision, illustrates its aggressive nature and tendency for local invasion and metastasis. The importance of close posttreatment monitoring cannot be overstated [19]. Regular follow-up with imaging and clinical assessments is crucial for early detection of recurrence. This allows for timely intervention and adjustments to the management plan, which may improve outcomes. Early recurrence detection also facilitates the use of salvage therapies and participation in clinical trials for novel treatments, offering additional options for patients with aggressive disease [18]. This underscores the necessity of considering malignancy in the differential diagnosis of pericardial masses and highlights the value of molecular and cytogenetic studies, such as SYT-SSX fusion analysis, for accurate diagnosis [17].

## Conclusions

In conclusion, while the prognosis for PSS remains poor, early detection, aggressive treatment, and regular follow-ups are critical for managing this disease. Aggressive treatment involves complete surgical resection, systemic chemotherapy, and consideration of targeted therapies or radiation therapy. Multidisciplinary care and advanced diagnostic tools, such as SYT-SSX fusion analysis, play a significant role in improving patient outcomes, particularly given the diagnostic challenges and potential for initial misdiagnosis. Ongoing research into novel therapies, including targeted inhibitors, immunotherapies, and biomarkers, is vital for advancing the treatment of this rare yet lethal malignancy.
